# Age related immune modulation of experimental autoimmune encephalomyelitis in PINK1 knockout mice

**DOI:** 10.3389/fimmu.2022.1036680

**Published:** 2022-11-17

**Authors:** Davide Cossu, Kazumasa Yokoyama, Shigeto Sato, Sachiko Noda, Tamami Sakanishi, Leonardo Antonio Sechi, Nobutaka Hattori

**Affiliations:** ^1^ Department of Neurology, Juntendo University, Tokyo, Japan; ^2^ Biomedical Research Core Facilities, Juntendo University, Tokyo, Japan; ^3^ Department of Biomedical Sciences, Division of Microbiology and Virology, University of Sassari, Sassari, Italy; ^4^ Division of Cell Biology, Juntendo University, Tokyo, Japan; ^5^ SC Microbiologia Azienda Ospedaliero Universitaria (AOU) Sassari, Sassari, Italy; ^6^ Neurodegenerative Disorders Collaborative laboratory, RIKEN Center for Brain Science, Saitama, Japan

**Keywords:** *PINK1*, neuroinflammation, experimental autoimmune encephalomyelitis, Parkinson’s disease, multiple sclerosis

## Abstract

**Objective:**

Recent research has shown that Parkin, an E3 ubiquitin ligase, modulates peripheral immune cells-mediated immunity during experimental autoimmune encephalomyelitis (EAE). Because the PTEN-induced putative kinase 1 (PINK1) protein acts upstream of Parkin in a common mitochondrial quality control pathway, we hypothesized that the systemic deletion of PINK1 could also modify the clinical course of EAE, altering the peripheral and central nervous systems’ immune responses.

**Methods:**

EAE was induced in female *PINK1^-/-^* mice of different age groups by immunization with myelin oligodendrocyte glycoprotein peptide.

**Results:**

Compared to young wild-type controls, *PINK1^-/-^* mice showed earlier disease onset, albeit with a slightly less severe disease, while adult *PINK1^-/-^* mice displayed early onset and more severe acute symptoms than controls, showing persistent disease during the recovery phase. In adult mice, EAE severity was associated with significant increases in frequency of dendritic cells (CD11C^+^, IAIE^+^), lymphocytes (CD8^+^), neutrophils (Ly6G^+^, CD11b^+^), and a dysregulated cytokine profile in spleen. Furthermore, a massive macrophage (CD68^+^) infiltration and microglia (TMEM119^+^) and astrocyte (GFAP^+^) activation were detected in the spinal cord of adult *PINK1^-/-^* mice.

**Conclusions:**

PINK1 plays an age-related role in modulating the peripheral inflammatory response during EAE, potentially contributing to the pathogenesis of neuroinflammatory and other associated conditions.

## Introduction

Mutations in the PTEN-induced putative kinase 1 (*PINK1)* gene, whose product is a kinase localized on the outer membrane of depolarized mitochondria, and in the *PARK2* gene, which encodes for the cytosolic E3 ubiquitin ligase Parkin, are implicated in autosomal recessive Parkinson’s disease (PD) (1). Both proteins are required for the maintenance of the mitochondrial quality knows as mitophagy, a process that involves the selective autophagic degradation of damaged mitochondria (2). Dysfunctional neuronal mitophagy has been linked to neuronal cell death in several neurodegenerative disorders, such as PD, Alzheimer’s disease, and multiple sclerosis (MS) (3). *PINK1^-/-^* and *Parkin^-/-^* mice do not exhibit a PD-relevant phenotypes, nor signs of neurodegeneration, and their serum cytokine profile remains unchanged (4, 5). However, the absence of these proteins worsens the acute inflammation caused by several stimuli, such as bacterial infection (6), lipopolysaccharide (7), or myelin oligodendrocytes glycoprotein- (MOG) induced pro-inflammatory responses (8). Recent work has demonstrated that an autoimmune response in the absence of PINK1 could participate in the etiology of PD following intestinal infection with Gram-negative bacteria in *PINK1^-/-^* mice, through mitochondrial specific CD8^+^ T- cells capable of killing dopaminergic neurons *in vitro* (6). Another study showed that Parkin can be a modulator of autoimmune inflammation in experimental autoimmune encephalomyelitis (EAE) (8), an animal model of inflammatory diseases of the central nervous system (CNS). EAE was worsened in *Parkin^-/-^* mice, which displayed an increasing number of CD8αβ^+^TCRαβ^+^ T-cells in both spleen and brain, as well as a strong overexpression of A1 reactive astrocytes induced by classical activated neuroinflammatory microglia in the spinal cord during the acute phase of the disease (8). Notably, different *Parkin^-/-^* mice that exhibited chronic disease progression displayed a reduced number of microglia, astrocytes, and oligodendrocytes in the midbrain (8).

In addition to the involvement of these proteins in the pathogenesis of PD, several studies have indicated a potential role for PINK1 and Parkin in the pathogenesis of MS, an autoimmune disease characterized by chronic inflammation, gliosis, and neuronal loss (9). Several cases of co-occurrence of PD after diagnosis of MS have been reported (10, 11), including a patient with early-onset PD and a heterozygous Parkin mutation who after 8 years developed primary progressive MS (12). In addition, various movement disorders have been described in patients with both early and progressive stages of MS (13).

Despite exhibiting different clinical profiles, common mechanism related to neurodegeneration such as mitochondrial dysfunctions, microglia activation, altered microbiota, and inflammation are observed in patients with PD and MS (14), suggesting converging pathogenic pathways of neurodegeneration.

In this study, EAE was induced with MOG_35-55_ peptide in *PINK1^-/-^* mice of different ages, to determine whether PINK1 plays a role in the modulation of peripheral and CNS immune responses, and to investigate the putative age-related mechanism involved.

## Method

### Mice

All experiments were performed in accordance with the Guidelines for Animal Experimentation of the Juntendo University School of Medicine (approved protocol no. 290238).

Young (7-8 weeks old) and adult (5-6 months old) female *PINK1^-/-^* and age-matched female wild-type mice (Charles River Laboratories Japan, Inc.) of the same C57BL/6J genetic background (N =20/group) were housed and maintained in a controlled environment at 22–24°C and 55% humidity, on 12 h light/dark cycles. with ad libitum access to food and water. Mutant mice lacking *PINK1^-/-^* were generated according to the protocol of Kitada et al. (5), and the mouse colony was maintained by intercrossing homozygotes.

### Induction of EAE

Active EAE was induced by immunization with 200 μg of MOG_35-55_ (MEVGWYRSPFSRVVHLYRNGK; BEX CO., LTD, Japan), emulsified in complete Freund’s adjuvant (4mg/mL), as previously described (8). Pertussis toxin was administered intraperitoneally on day 0 and 48 h after immunization (200 ng/injections). A control group of age-matched littermate females were injected subcutaneously with an emulsion of phosphate-buffered saline (PBS) in complete Freund’s adjuvant, and with the same concentration of pertussis toxin. All animals were observed and weighed daily and scored using a five-point clinical scoring system: 0 = no clinical symptoms, 1 = paralyzed tail, 2 = partial hind limb paresis, 3 = paralysis of both hind limb, 4 = forelimbs paralysis, and 5 = moribund state or death.

### Lymphocytes proliferation assay

The MojoSort Mouse CD4 or CD8 T-Cell Isolation Kit (BioLegend) was used for the isolation and purification of CD8^+^ or CD4^+^ T-cells from the spleen of EAE mice through immunomagnetic negative selection. For the T-cell proliferation assay, unfractionated, CD4^+^ or CD8^+^ responder T-cells were cultured for 2 days with 50 μg/mL MOG_35-55_ or with 5 μg/mL phytohemagglutinin or with 50 μg/mL ovalbumin as positive and negative controls, in the presence of gamma-irradiated (3,000 rad) accessory spleen cells syngeneic to the responding T-cells at 1 × 10^6^ cells/mL, as previously published (8). Cell proliferation during the last 18 h was determined by measuring the radioactivity of the incorporated ^3^H-thymidine (1μCi/well) (PerkinElmer, Waltham, MA, USA) using a microplate scintillation counter (MicroBeta TriLux, PerkinElmer). The proliferative response was expressed as a stimulation index (SI): counts per minute (cpm) of stimulated cells/cpm of unstimulated cells.

### Histology and immunohistochemistry

Mice were euthanized by intraperitoneal injection of sodium pentobarbital overdose during the acute phase (day 15) of EAE, and transcardially perfused with 4%-paraformaldehyde (Nacalai Tesque, Kyoto, Japan). Once perfused, the entire spinal columns were removed and placed in 4%-paraformaldehyde for 2 days at 4°C for immersion fixation. The spines were sectioned and tissues were embedded in paraffin, and consecutive sections at 6 μm thickness were cut for subsequent staining. Hematoxylin/eosin and Klüwer-Barrera staining were performed on all sections to detect inflammatory cell infiltration and demyelination, respectively. Spinal cord demyelination was evaluated by measuring the percentage of demyelinated area in relation to the white matter volume, while the degree of inflammation was expressed as the percentage of the infiltrated area over the total spinal cord sections.

Immunohistochemistry was performed on paraffin-embedded spinal cord sections as previously reported (15). Protein expression on tissue was evaluated with primary antibodies raised against for CD68 (1:500), glial fibrillary acidic protein (GFAP) (1:2000), transmembrane protein 119 (TMEM119) (1:300), CD45 (1:50), tubulin polymerization-promoting protein (TPPP) (1:100), and CD3 (1:1000) (all purchased from Abcam, Tokyo, Japan), followed by incubation with the appropriate secondary antibodies. Positive cells were visualized as brown using the 3,3′-Diaminobenzidine (Vector Laboratories, USA) and counterstained with hematoxylin. Images were analyzed using Olympus CellSens software (Olympus, Tokyo, Japan). The results were expressed by modifying a previously published semi-quantitative score using the following scale: 0 = none, 1 = low expression or less than 5 positive cell/microscope field, 2 = moderate expression or 5 to 10 positive cell/microscope field, 3 = high expression or 15 to 20 positive cell/microscope field, 4 = very high expression or more than 20 positive cells/microscope field, and 5 = more than 60 positive cells/microscope field (8, 15).

### Flow cytometry

Single-cell suspensions of spleen cells (1 × 10^6^ cells) were stained with live/dead marker (Zombie NIR Fixable Viability Kit, Biolegend, USA) for 15 min at room temperature (24°C), then preincubated with FcBlock (FcγRII-RIII) for 10 min at 4°C. For flow cytometry analysis, cells were stained, for 20 min on ice, with APC anti-CD3 (clone 17A2), FITC anti-CD4 (clone GK1.5), BV 421 anti-CD8a (clone 53-6.7), PerCP/Cy5.5 anti-CD19 (clone 1D3), Alexa Fluor 488 anti-CD11c (clone N418), BV 510 anti-I-A/I-E (clone M5/114.15.2), PE/Cy7 anti-CD11b (clone M1/70), Alexa Fluor 700 anti-Ly6G (clone 1A8), and PE anti-CD115 (clone AFS98) (all purchased from Biolegend), washed thoroughly and resuspended in 400 μL of fluorescence-activated cell sorting (FACS) buffer. All samples were acquired (20000 events/sample) on BD FACSCelesta™ (BD Biosciences, USA), and data were analyzed using the FlowJo software v10.8.1 (Tree Star, Ashland, OR, USA).

### Cytokine expression upon antigen-specific T-cell stimulation

Spleen cells were isolated from all mice at the acute phase (day 15) and incubated with MOG_35-55_ peptide for 48 h, as previously reported (8). A Multi-Analyte ELISArray kit (Qiagen, Hilden, Germany) was used to detect the concentration of several inflammatory cytokines from the culture supernatants according to the manufacturer’s protocol.

### Statistics

Statistical analysis was performed using GraphPad Prism v9.3.1 (GraphPad Software, La Jolla, CA, USA). Clinical scores were analyzed by the Mann-Whitney nonparametric u-test. When comparing EAE scores and body weights between groups, two-way repeated measures analysis of variance (ANOVA) was performed followed by *post-hoc* Bonferroni’s test for multiple comparisons. A one-way ANOVA followed by *post-hoc* Dunnett’s multiple comparison test was used to evaluate the histological and immunohistochemical analysis of the spinal cords. Data obtained with flow cytometry, T-cell proliferation, and ELISA assays, were analyzed with a two-tailed Student’s t-test for pairwise comparisons. Results are expressed as mean ± standard error mean for parametric tests, or median and interquartile range for non-parametric tests. Statistical significance was considered when *p* < 0.05.

## Results

### Clinical assessment of EAE

*PINK1^-/-^* mice with EAE exhibited a specific disease-related phenotype, with atypical clinical symptoms such as rigidity and repeated muscle spasms. Moreover, *PINK1^-/-^* mice showed enhanced aggressive behavior compared to control mice without clinical symptoms.

**Figure 1:**
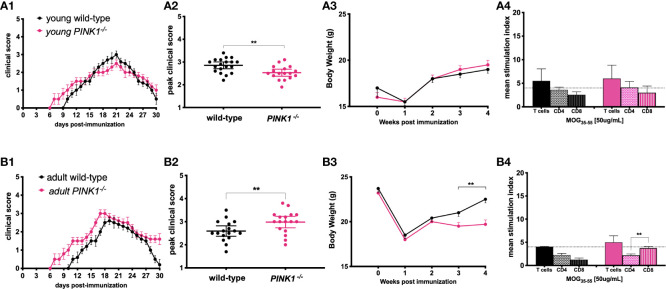
EAE development in *PINK1^-/-^* mice. Graph reports EAE mice clinical score evaluated daily **(A1, B1)**, the peak clinical scores **(A2, B2)**, body weight **(A3, B3)**, and T-cell proliferative response to MOG_35–55_ peptide **(A4, B4)**. The data show one experiment (N =10 mice/group) of three independent ones for the proliferation assay. Data are expressed as means ± SD and mean with 95% confident interval calculated by ANOVA followed by Bonferroni’s posthoc test or Mann-Whitney u test. *p < 0.0, **p < 0.01, ***p < 0.001.

Young *PINK1^-/-^* mice displayed an early onset but reduced EAE severity compared to the wild-type controls (**Figure 1A1**). There was no change in the EAE recovery phase in either young wild-type and *PINK1^-/-^* mice (**Figure 1A1**). Instead, the knockout of *PINK1* in adult mice had an effect on severity, day of onset, and recovery phase (**Figure 1B1**). Mean disease peak severity was significantly higher in young wild-type mice as compared with young *PINK1^-/-^* mice (**Figure 1A2**); however, adult *PINK1^-/-^* mice showed the highest peak of disease among them (**Figure 1B2**). Body weight loss was similar between young *PINK1^-/-^* and control mice during EAE (**Figure 1A3**), while it was significantly decreased in adult *PINK1^-/-^* mice having no symptom recovery (**Figure 1B3**). Overall, *PINK1^-/-^* mice showed a gradual increase in clinical symptoms with increasing age and, in particular, an absence of a recovery phase in adult mice. **Table 1** illustrates the summary of the incidence, mortality, and severity that resulted from active EAE immunization.

**Table 1:** Age-related differences on disease course in MOG_35-55_-induced EAE.

Mouse strain, and age		Geneticmutation		EAE incidence ^a^	Mortality ^b^	Mean day of onset ^c^	Peak clinical score ^d^
C67BL/6J (7-8 weeks)	wild type		95%		0%	10.3 ± 0.6	2.8 ± 0.3
C67BL/6J (7-8 weeks)	*PINK1^-/-^*		85%		10%*	7 ± 1.0*	2.5 ± 0.5
C67BL/6J (5**-**6 months)	wild type		90%		5%	11 ± 0.5	2.6 ± 0.4
C67BL/6J (5-6 months)	*PINK1^-/-^*		90%		5%	7 ± 1.7*	3.0 ± 0.3*

*Statistically significant (^a-b^ χ2 test; ^c–d^ Student’s t-test).

### T-cell proliferation during the acute phase of EAE

The percentage of CD3^+^ T cells in spleen ranged from mean values of 25% to 30% in young and adult MOG-primed animals. Preliminary assays indicated that MOG_35-55_ at the final concentration of 50 μg/mL had the strongest stimulatory effect on both CD8^+^ and CD4^+^ T-cells. In young wild-type mice with EAE, there were no significant differences in T-cell proliferation between wild-type and *PINK1^-/-^* mice (**Figure 1A4**). Conversely, in adult mice with EAE, MOG_35-55_ stimulation increased the proliferation of T lymphocytes, with a significantly (*p* = 0.045) stronger stimulatory effect on CD8^+^ T-cells than on CD4^+^ T-cells (**Figure 1B4**).

### Histological and immunohistochemical analysis of the spinal cord

**Figure 2:**
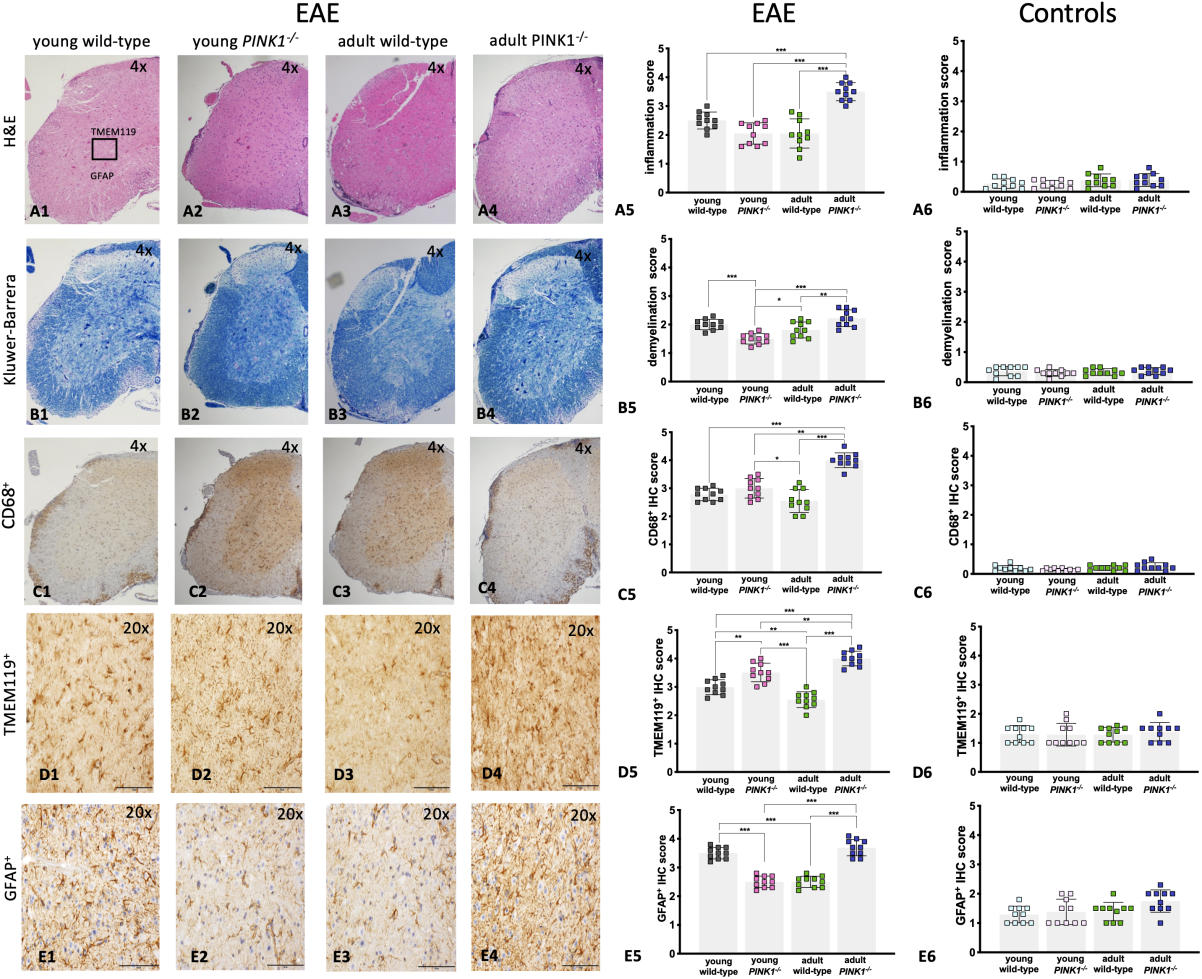
Histological and immunohistochemical analysis of the spinal cord tissue among groups during the EAE acute phase. **(A1–A4)** histological images of inflammatory cell infiltration (scale bar = 200 um); **(B1–B4)** extent of demyelination determined by Klüver-Barrera staining (scale bar = 200 um); **C1–C4** images of infiltrated CD68^+^ macrophages/monocytes in the white matter (scale bar = 200 um); **(D1–D4)** images of TMEM119^+^ microglia expression in the gray matter (scale bar = 100 um); **(E1–E4)** images of GFAP^+^ astrocytes expression in the gray matter (scale bar = 100 um); for the semi quantitative analysis of EAE mice **(A5–E5)** and no immunized controls (A6-E6), stain-positive cells in the lumbar spinal cord were counted in two randomly chosen sections for five mice of each group. Data were analyzed using one-way ANOVA followed by *post-hoc* Dunnett’s multiple comparison test. Each symbol represents an individual mice/*p <0.0, **p < 0.01, ***p < 0.001.

Hematoxylin/eosin (**Figures 2A1–A6**) and Klüwer-Barrera (**Figures 2B1–B6**) staining of longitudinally sectioned spinal cord sections (cranial, thoracic, lumbar, sacral) revealed the multifocal nature of the lesions associated with EAE, showing few lesions in some area, while others with variable degree of inflammation and demyelination. In general, the most severe lesions were observed in adult *PINK1^-/-^* mice (**Figures 2A4, B4**).

Immunohistochemical staining was performed on longitudinal sections to identify key immune cells within the EAE spinal cord lesions. A high number of infiltrating macrophages (CD68^+^) was detected in adult *PINK1^-/-^* mice, whereas there was no statistical difference between young *PINK1^-/-^* and wild-type mice (**Figures 2C1-C6**). Microglia (TMEM119^+^) expression in the gray matter of the spinal cord was higher in both young and adult *PINK1^-/-^* mice, as compared to the wild-type counterparts (**Figures 2D1–D6**), with the highest immunohistochemistry (IHC) score observed in adult *PINK1^-/-^* mice. Enhanced astrocyte (GFAP^+^) expression was found in the white and gray matter of the spinal cord of young wild-type and adult *PINK1^-/-^* mice that developed the most severe disease (**Figures 2E1-E6**). Finally, there was no statistical difference between the groups in terms of T-cell (CD3^+^) infiltration.

### Peripheral cells and cytokine profiles

**Figure 3:**
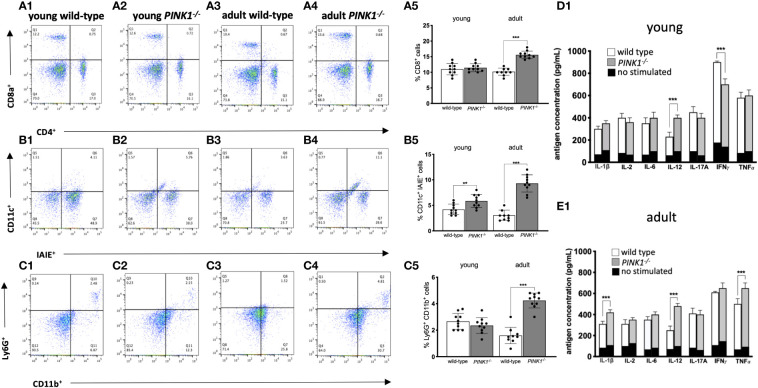
Immune cell populations and cytokine profiles in splenocytes during the acute phase. **(A1–A5)** Representative blots showing CD4^+^ and CD8^+^ T lymphocytes and its representative graph. **(B1–B5)** Representative blots showing conventional dendritic cells and its representative graph. **(C1–C5)** Representative blots showing different myeloid cells and its representative graph. Cytokine expression profiles in culture supernatants of MOG_35-55_-stimulated spleen cell **(D1, E1)**. The data show one experiment (N =10 mice/group) of three independent ones. Data are expressed as mean ± SD calculated using Student’s t-test *p <0.0, **p < 0.01, ***p < 0.001.

Young *PINK1^-/-^* mice had a significantly (*p* < 0.0001) high number of lymphocyte antigen 6 complex locus G (Ly)6G^-^CD11b^+^ myeloid cells (primarily monocytes and macrophages) than young controls (**Figures 3C1, C2**) in the spleen at the peak of EAE. Young *PINK1^-/-^ mice* also showed a higher number of splenic dendritic cells (DCs) (IAIE^+^ CD11c^+^) than young controls during the acute phase (**Figures 3B1, B2, B5**).

Adult *PINK1^-/-^* mice showed a significantly (*p* < 0.0001) higher percentage of T-cells (CD8^+^) (**Figures 3A1–A5**) as well as DCs (**Figures 3B1–B5**) in comparison to adult wild-type controls during the acute phase. Adult *PINK1^-/-^* mice also displayed a significantly (*p* < 0.0001) higher percentage of neutrophils (Ly6G^+^CD11b^+^) compared to adult wild-type and to both young *PINK1^-/-^* and wild-type mice (**Figures 3C3-C5**).

All EAE mice showed a reduced number of B-cells (CD19^+^) than nonimmunized mice, regardless of age or genetic background (data not shown).

Analysis of the cytokine profile in spleen cells from young mice with EAE showed a significant (*p* < 0.0001) increase in the level of interferon gamma (IFN-γ) in wild-type mice and interlukin-12 (IL-12) in PINK1^-/-^ mice in response to MOG_35-55_ peptide (**Figure 3D1**). In adult mice with EAE, PINK1 deficiency highly (*p* < 0.0001) increased the expression of IL-6, IL-12, and tumor necrosis factor-α (TNF-α) (**Figure 3E1**).

## Discussion

Our data provide evidence that an abnormal peripheral immune response occurs in the absence of PINK1 during EAE and is associated with age-related changes in the CNS immune system. Overall, adult *PINK1^-/-^* mice developed an earlier onset and more severe disease compared to wild-type controls and young *PINK1^-/-^* mice. The increased severity of EAE symptoms was strongly associated with a higher number of peripheral CD8^+^ T-cells, DCs, neutrophils, and a dysregulated inflammatory cytokine profile, together with macrophage infiltration and strong microglia-astrocyte activation in the spinal cord.

The results of this study partially agree with those reported previously by our group, which demonstrate that the deletion of Parkin, a protein that acts downstream of PINK1 in the same genetic pathway, causes an enhancement of EAE severity (8). Consistent with this, young *Parkin^-/-^* mice with severe EAE showed a high expression of CD8^+^ T-cells in both the spleen and brain, a robust macrophage infiltration and microglia activation in the spinal cord (8). Furthermore, these findings are in line with previous research, which provided evidence that intestinal infection in *PINK1^-/-^* mice elicits the recruitment of cytotoxic CD8^+^ T-cells in the periphery and in the brain, triggering PD-like symptoms (6). One of the proposed mechanisms was that PINK1and Parkin regulate the immune response acting on the major histocompatibility (MHC) class I antigen presentation pathway, inhibiting mitochondrial antigen presentation (16). Oligoclonal T-cell expansion involves predominantly CD8^+^ T-cells in CNS lesions of patients with MS (17), however, it is unclear whether these cells play a pathogenic or regulatory role. In EAE mice, most of the clonally expanded CD8^+^ T-cells that did not respond to myelin antigen correspond to regulatory CD8^+^ T-cells (17). Conversely, some studies have shown another population of myelin-specific pathogenic CD8^+^ T-cells, which play a proinflammatory role during EAE initiated by CD4^+^ T-cells (18). Hence, different CD8^+^ T-cells subtypes may exert different functions and exhibit different properties in MS and EAE.

Despite the common features in the development of EAE, there are also several key differences between *PINK1^-/-^* and *Parkin^-/-^* mice. First, in our system the systemic deletion of PINK1 affected the peripheral recruitment of DCs, especially in adult *PINK1^-/-^* mice with EAE. Myeloid cells, such as DCs, are recognized as playing a key role in the effector phase of EAE (19, 20), even if the exact mechanism of the trafficking of DCs across the blood-brain barrier still needs to be characterized. Moreover, the priming of MHC class I-restricted CD8^+^ T-cells is DC-dependent in CNS inflammation (21). In addition, the increased expression of pathogenic T-cell cytokines in EAE, such as IL-1β, IL-12, and TNF-α, detected in *PINK1^-/-^* mice, might leave the CNS more vulnerable to inflammation (22). Interestingly, the levels of IL-17A and IFN-γ, two cytokines that play a critical role in the pathogenesis of EAE, were unchanged in the spleen of young and adult *PINK1^-/-^* mice, suggesting that the lack of PINK1 may not affect the functional response of these proinflammatory mediators during the progression of EAE.

Second, PINK1 deletion had a stronger impact on EAE in adult mice, indicating that relatively narrow age ranges associated with mitochondrial dysfunction, can produce significant differences not only in the behavior (23), but also in immune-mediated mechanisms during adulthood in C57BL6/J mice. It should be noted that EAE severity increases with age in C57BL/6J mice (6-8 months) (24), however, in the present study no significant differences were found between young and adult wild type mice. A possible reason behind this result could be that we used adult mice ranging in age from 5-6 months, not enough to modify EAE progression. Mice are considered adult after 8 weeks; however, rapid growth for most biological processes is observed until 3 months of age, while past 6 months, mice might be affected by senescence (25). It is acknowledged that deficient mitophagy increases with aging (26), which is one of the main risk factors for the development of many neurodegenerative diseases, characterized by a progressive increase in neuroinflammation (27). Although there are currently no data that directly compare the impact of Parkin and PINK1 deficiency on EAE development between mature adult (3–6 months old), middle-aged (10–14 months old), and old (18–24 months old) mice, in our study, *PINK1^-/-^* mice seemed to follow this trend, with the severity of disease increasing with age.

Furthermore, all *PINK1^-/-^* mice displayed an earlier disease onset compared to age-matched wild-type controls. During the pre-symptomatic phase of EAE, blood-brain barrier breakdown plays an essential role in disease pathogenesis by allowing immune cells and mediators to reach the CNS (24). Based on our findings, we hypothesize that PINK1 deletion and defective neuronal mitophagy, might modulate the early effector phase of EAE by promoting alteration in the blood-brain barrier permeability (28).

Interestingly, in *PINK1^-/-^* and *Parkin^-/-^* mice upon acute mitochondrial stress, the activation of stimulator of interferon genes (STING) led to a robust inflammatory phenotype (29). However, a STING agonist seems to attenuate EAE *via* type I IFN-dependent and independent pathways (30). We can suppose that the inflammatory EAE phenotypes associated with loss of PINK1 or Parkin are not always due to abnormal activation of the STING signaling pathway (31), indicating that defective mitochondria promote or dampen neuroinflammation and that inflammation itself contributes to mitochondrial dysfunction (32).

In summary, our data demonstrated that mutations in the *PINK1* gene might contribute to the CNS inflammation, affecting peripheral and glia-dependent immune responses. Furthermore, our data suggest that DCs may play a key role in promoting the activation of microglia and astrocytes.

Additional research should be carried out to better understand the effect of PINK1 especially on the effector phase of EAE, by using the adoptive transfer model in mice with conditional deletion of PINK1 in neurons. Moreover, future research could explore the functional role of these aging-induced DCs on neuroinflammation, to discover new DCs-based druggable targets and treatment opportunities for neuroinflammatory conditions. Finally, the development of new animal models is needed to fully understand the mechanisms and implications of these findings for human diseases.

## Data availability statement

The raw data supporting the conclusions of this article will be made available by the authors, without undue reservation.

## Ethics statement

All experiments were performed in accordance with the Guidelines for Animal Experimentation of the Juntendo University School of Medicine (approved protocol no. 290238).

## Author contributions

DC designed and performed experiments, analyzed data, and wrote the manuscript; KY analyzed data, and provided feedback on the manuscript; SS and SN provided knockout mice for experiments; TS supervised the flow cytometry experiments; LS and NH critically revised the draft and approved the final manuscript. All authors contributed to the article and approved the submitted version.

## Funding

This work was supported by JSPS KAKENHI (grant number: JP 20K16468) and by UNISS DSBM 2022 to DC.

## Acknowledgments

We thank the Laboratory of Molecular and Biomedical Research, and the Laboratory of Morphology and Image Analysis, Biomedical Research Core Facilities, Juntendo University Graduate School of Medicine, for technical assistance.

## Conflict of interest

The authors declare that the research was conducted in the absence of any commercial or financial relationships that could be construed as a potential conflict of interest.

## Publisher’s note

All claims expressed in this article are solely those of the authors and do not necessarily represent those of their affiliated organizations, or those of the publisher, the editors and the reviewers. Any product that may be evaluated in this article, or claim that may be made by its manufacturer, is not guaranteed or endorsed by the publisher.
